# Relationship between fibrinogen level and advanced colorectal adenoma among inpatients: A retrospective case-control study

**DOI:** 10.3389/fmed.2023.1140185

**Published:** 2023-03-16

**Authors:** Huijie Wang, Huanwei Zheng, Xu Cao, Ping Meng, Jinli Liu, Zhichao Wang, Teng Zhang, Haiying Zuo

**Affiliations:** ^1^Department of Endoscopy, Shijiazhuang Traditional Chinese Medicine Hospital, Shijiazhuang, China; ^2^Department of Gastroenterology, Shijiazhuang Traditional Chinese Medicine Hospital, Shijiazhuang, China; ^3^Graduate School, Hebei North University, Zhangjiakou, China; ^4^Institute of Traditional Chinese Medicine, North China University of Science and Technology, Tangshan, China

**Keywords:** association, fibrinogen, advanced colorectal adenoma, retrospective study, case-control study

## Abstract

**Objective:**

This study was to explore the relationship between fibrinogen and advanced colorectal adenoma among inpatients.

**Methods:**

From April 2015 to June 2022, 3738 participants (566 case subjects and 3172 control subjects) who underwent colonoscopies enrolled, and smooth curve fitting and logistic regression models were applied to explore the association between fibrinogen and advanced colorectal adenoma. In addition, sensitivity and subgroup analyses were performed to assess the stability of the results.

**Results:**

Compared with lower fibrinogen quantile 1 (< 2.4 g/L), the adjusted OR values for fibrinogen and advanced colorectal adenoma in quantile 2 (2.4–2.75 g/L), quantile 3 (2.76–3.15 g/L), and quantile 4 (≥3.16 g/L) were 1.03 (95% confidence interval [CI]: 0.76–1.41), 1.37 (95% CI: 1.01–1.85), and 1.43 (95% CI: 1.06–1.94), respectively. A linear relationship between fibrinogen and advanced colorectal adenoma was observed. Sensitivity and subgroup analyses showed stable results.

**Conclusion:**

Complements the evidence that fibrinogen was positively associated with advanced adenomas, suggesting that fibrinogen may play a role in the adenoma-carcinoma sequence.

## 1. Introduction

A colorectal adenoma is the precursor to colorectal cancer (CRC) (1). The adenoma-carcinoma sequence is central to the pathogenesis of CRC (2). Although most CRCs originate from colorectal adenoma (3–5), most common adenomas cannot develop into cancer (5, 6), and advanced colorectal adenomas are instead more likely to become cancerous.

Some coagulation indicators have been recognized as potential biomarkers for CRC (7–10). However, research on risk factors for advanced adenomas, a critical stage in the adenoma-carcinoma pathway, is limited (11–13), especially for the components of the hemostatic system (14–16).

Fibrinogen is a glycoprotein produced in the liver (10). The interaction of fibrinogen with the perivascular environment influences the progression of the disease beyond its conventional role in the acute hemostatic cascade and is associated with the disease that has inflammatory components, particularly with pro-inflammatory effects in several types of cancer (17). The relationship between fibrinogen and CRC has been studied. Preoperative elevated fibrinogen levels were associated with poor prognosis/disease-free survival and worst response to treatment and tumor size (18–21). Based on the theory of adenoma-carcinoma sequence and the existing studies suggesting that fibrinogen may influence the progression from adenoma to CRC, we hypothesized that fibrinogen is associated with advanced adenoma.

Electronic medical records generate a wealth of clinical data that can describe conditions in detail and are the hallmark of modern healthcare systems. Clinical data can be extracted and integrated, cleaned and transformed, and converted into a data format to create a database that can be used for disease management, early diagnosis, or treatment decisions (22).

Studies targeting advanced colorectal adenoma are more critical to understand the adenoma-carcinoma sequence (12) and be beneficial for developing strategies to prevent CRC (12). Given that the association between fibrinogen plasma levels and advanced adenoma has not been studied, we conducted this case–control study to assess the association between fibrinogen plasma levels and advanced adenoma.

## 2. Materials and methods

### 2.1. Study population

The present study was conducted among 3,738 consecutive inpatients who underwent colonoscopies from April 2015 to June 2022. Subjects with advanced colorectal adenoma who underwent pathological examination to confirm the diagnosis were considered eligible cases. Subjects with normal colonoscopies during the same period were considered controls. Each patient was analyzed only once.

**Figure 1:**
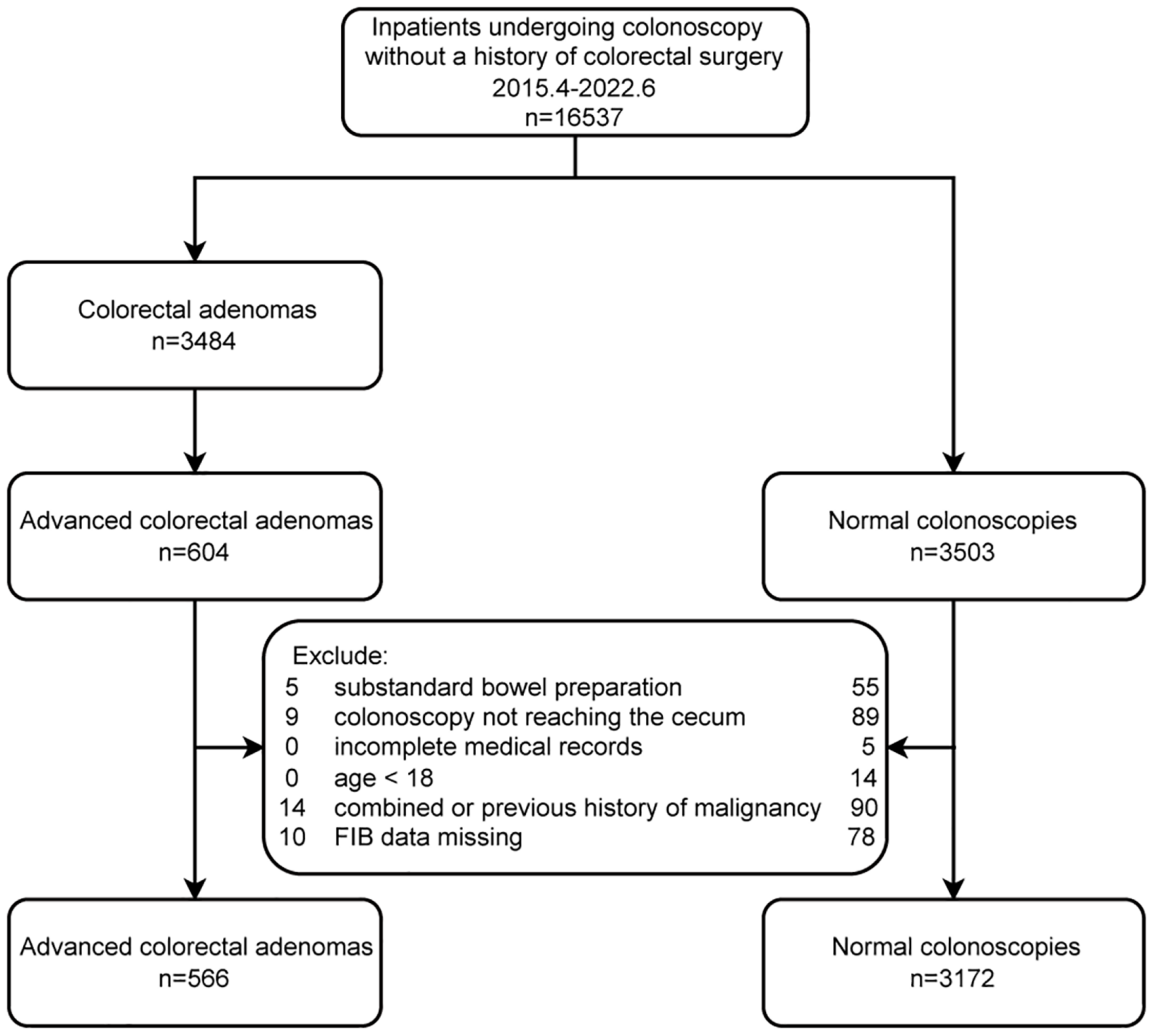
Flow diagram of the screening and enrollment of study participants.

Exclusion criteria: history of colorectal surgery, an incomplete colonoscopy (no cecum reached), inadequate bowel preparation, younger than 18, incomplete medical records, history of any cancer treatment within 3 years, combined with malignancies, and fibrinogen data missing. A total of 3,738 subjects (566 cases and 3,172 controls) were included. Shown in Figure 1.

### 2.2. Definition of results and indicators

For the current study, individuals with advanced colorectal adenoma, defined in another piece of literature (23), comprised the case group.

Plasma fibrinogen was measured using a Mindray CX-9000 automatic coagulation analyzer (Shenzhen, China) and its associated reagents. As soon as possible after collection, all blood samples were processed within 4 h. In the present study, the median time between colonoscopy and fibrinogen testing is 2 (1, 5) days.

The baseline variables were extracted from electronic medical records, including age, sex, weight, marital status, past medical history, family history (including colorectal and digestive system malignancy), drinking status, smoking status, and co-morbidities. Marital status is divided into three categories: married, single/divorced, and other. The history of smoking and drinking were classified into four categories, respectively: never, former, current, and NA (not recorded). A current drinker is defined as an individual who consumes any type of alcohol per week (self-reported) (24). An ex-smoker or ex-drinker is defined as someone who has smoked or used alcohol in the previous period, although the smoking has now ceased. Co-morbidities included hypertension, ischemic cerebrovascular disease, coronary heart disease (CHD), hyperlipidemia (HLP), liver diseases (including liver cirrhosis, fatty liver, and hepatitis), and diabetes mellitus (DM). Individuals with DM were diagnosed with one of the following criteria: HbA1c ≥6.5%, fasting glucose ≥7.0 mmol/L (25, 26), or medical records. In addition, we extracted the data of participants from the laboratory information system and electronic medical records during their hospitalization, including fibrinogen, albumin (ALB), aspartate aminotransferase (AST), alanine aminotransferase (ALT), glucose (GLU), alkaline phosphatase (ALP), creatinine (CREA), urea, uric acid (UA), platelets (PLT), thrombin time (TT), activated partial thromboplastin time (APTT), the international normalized ratio of prothrombin time (PT-INR), prothrombin time (PT), activated partial thromboplastin time (APTT). If there are multiple eligible test reports for the same test during the hospitalization period, the first result within this period of hospitalization will prevail (before the colonoscopy).

### 2.3. Statistical analysis

Continuous variables are expressed as mean ± standard deviation or median (quartile 1–quartiles 3) values according to the normality of the distribution. Comparisons between groups were performed by the chi-squared test or Fisher exact test and Student’s *t*-test or the Mann–Whitney *U*-test for categorical variables or continuous variables, respectively, as appropriate.

We evaluated the relationship between fibrinogen and advanced colorectal adenoma using smooth curve fitting and logistic regression analysis. To evaluate the impact of fibrinogen, fibrinogen level was categorized into quartiles (quartile 1, <2.40 g/L; quartile 2, 2.40–2.75 g/L; quartile 3, 2.76–3.15 g/L; quartile 4, ≥3.16 g/L). We constructed three models: (i) crude model, no other covariates were adjusted; (ii) adjustment by age and sex; and (iii) adjustment by age, sex, hypertension, DM, APTT, PLT, ALP, and CREA. These potential confounders were chosen based on previous scientific literature, or a more than 10% change in effect estimates. Subgroup analyses were performed using a binary logistic regression model and then performed an interaction test. In the subgroup analyses, considering the small number of ex-smokers and ex-drinkers, we integrated ex-smokers and ex-drinkers with non-smokers and non-drinkers as a group, respectively. Multiple imputations (five replications), based on a chained equation approach method in the R mice procedure, were performed to maximize statistical power and reduce bias to account for missing data. In addition, sensitivity analysis was conducted using all complete cases.

All statistical analyses were conducted by packages R 3.3.2 (http://www.R-project.org, The R Foundation) and Free Statistics software version 1.7.1, *p* < 0.05 (two-tailed test) was considered statistically significant.

The retrospective study was approved by the Ethics Review Board of Shijiazhuang Traditional Chinese Medicine Hospital (NO.20220919029). Requirements for informed consent were waived due to the retrospective nature.

## 3. Results

### 3.1. Study population characteristics

A total of 3,738 subjects, including 566 subjects with advanced colorectal adenoma and 3,172 with normal colonoscopies, were included in this study. The subjects’ baseline characteristics were shown in Table 1. Some differences existed between groups concerning various covariates, including age, sex, weight, smoking status, drinking status, fibrinogen, ALB, ALP, UA, GLU, CREA, Urea, PLT, hypertension, ischemic cerebrovascular disease, CHD, DM (all *p* < 0.05).

**Table 1:** Baseline characteristics of participants.

Variables	Total	Case	Control	*p*-value
	*n* = 3,738	*n* = 566	*n* = 3,172	
Age, (year)	52.3 ± 13.0	61.4 ± 10.2	50.7 ± 12.7	**<0.001**
Sex, male, *n* (%)	1,559 (41.7)	366 (64.7)	1,193 (37.6)	**<0.001**
Marital status, *n* (%)				0.105
Single/divorced	160 (4.3)	145 (4.6)	15 (2.7)	
Married	3,376 (90.3)	2,854 (90)	522 (92.2)	
Others	202 (5.4)	173 (5.5)	29 (5.1)	
Weight, (kg)	67.2 ± 12.4	70.6 ± 11.8	66.6 ± 12.4	**<0.001**
Smoking status, *n* (%)				**<0.001**
Non-smoker	2,331 (62.4)	317 (56)	2014 (63.5)	
Current smoker	142 (3.8)	52 (9.2)	90 (2.8)	
Ex-smoker	28 (0.7)	7 (1.2)	21 (0.7)	
NA	1,237 (33.1)	190 (33.6)	1,047 (33)	
Drinking status, *n* (%)				**<0.001**
Non-drinker	2,319 (62.0)	317 (56)	2002 (63.1)	
Current drinker	181 (4.8)	55 (9.7)	126 (4)	
Ex-drinker	17 (0.5)	5 (0.9)	12 (0.4)	
NA	1,221 (32.7)	189 (33.4)	1,032 (32.5)	
FIB, (g/L)	2.9 ± 0.7	3.0 ± 0.8	2.8 ± 0.7	**<0.001**
ALB, (g/L)	44.3 ± 3.7	43.7 ± 4.1	44.5 ± 3.6	**<0.001**
ALT, (U/L)	17.0 (13.0, 26.0)	18.0 (13.0, 25.0)	17.0 (13.0, 26.0)	0.071
AST, (U/L)	19.5 (16.9, 24.0)	19.2 (17.0, 23.0)	19.6 (16.7, 24.0)	0.777
ALP, (U/L)	75.1 ± 24.6	80.4 ± 24.9	74.1 ± 24.5	**<0.001**
UA, (μmol/L)	303.3 ± 94.5	322.7 ± 90.3	299.8 ± 94.8	**<0.001**
GLU, (mmol/L)	6.0 ± 1.7	6.4 ± 2.0	5.9 ± 1.7	**<0.001**
CREA, (μmol/L)	64.0 ± 21.6	71.4 ± 38.2	62.6 ± 16.6	**<0.001**
Urea, (mmol/L)	4.8 ± 1.5	5.1 ± 1.9	4.7 ± 1.4	**<0.001**
PLT, (×10^9^/L)	236.1 ± 60.2	223.5 ± 60.4	238.4 ± 59.9	**<0.001**
APTT, (s)	29.3 ± 5.5	29.0 ± 5.8	29.4 ± 5.5	0.109
TT, (s)	16.3 ± 1.8	16.2 ± 1.7	16.4 ± 1.8	0.104
PT-INR	1.0 ± 0.1	1.0 ± 0.1	1.0 ± 0.1	0.989
PT, (s)	12.0 ± 1.1	12.0 ± 1.1	12.0 ± 1.1	0.621
Family history, *n* (%)				
Colorectal cancer	54 (1.4)	11 (1.9)	43 (1.4)	0.28
Digestive system malignancy	189 (5.1)	34 (6)	155 (4.9)	0.262
Co-morbidities, *n* (%)				
Hypertension	926 (24.8)	234 (41.3)	692 (21.8)	**<0.001**
Ischemic cerebrovascular disease	370 (9.9)	72 (12.7)	298 (9.4)	**0.015**
CHD	419 (11.2)	89 (15.7)	330 (10.4)	**<0.001**
HLP	357 (9.6)	59 (10.4)	298 (9.4)	0.443
Previous History of cancer	59 (1.6)	14 (2.5)	45 (1.4)	0.064
Liver disease	402 (10.8)	51 (9)	351 (11.1)	0.146
DM	640 (17.1)	158 (27.9)	482 (15.2)	**<0.001**

Data presented are mean ± SD, median (Q1–Q3), or *N* (%).

FIB, fibrinogen; ALB, albumin; ALT, alanine aminotransferase; AST, aspartate aminotransferase; ALP, alkaline phosphatase; UA, uric acid; GLU, glucose; CREA, creatinine; PLT, platelets; APTT, activated partial thromboplastin time; TT, thrombin time; PT-INR, international normalized ratio of prothrombin time; PT, prothrombin time; CHD, coronary heart disease; HLP, hyperlipemia; DM, diabetes mellitus; and NA, not recorded. Bold values indicate statistical significance.

### 3.2. Effects of fibrinogen on advanced colorectal adenoma

**Figure 2:**
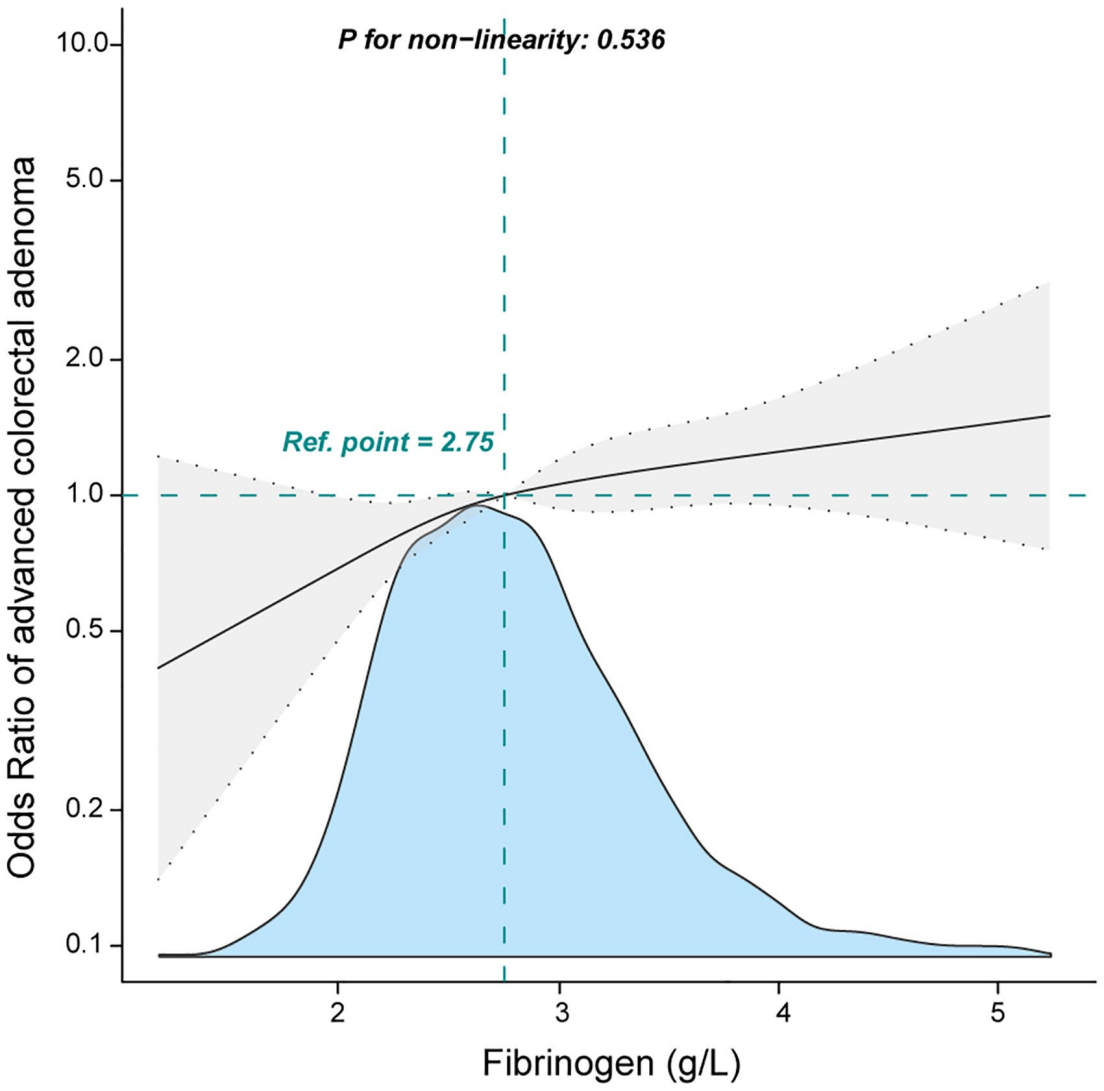
Association between fibrinogen and advanced colorectal adenoma among inpatients. They were adjusted for age, sex, albumin, alkaline phosphatase, creatinine, platelets, activated partial thromboplastin time, hypertension, and diabetes mellitus.

In multivariable logistic regression analyses, a positive relationship was found between fibrinogen and advanced colorectal adenoma. In the crude model, fibrinogen was found to be positively related to advanced colorectal adenoma [odds ratio (OR), 1.36; 95% confidence interval (CI), 1.21–1.53]. The results were similar after adjusting for age and gender (OR, 1.17; 95% CI, 1.03–1.33). After adjustment for sex, age, hypertension, DM, APTT, PLT, CREA, ALP, and ALB, the OR value was also stable (OR, 1.14; 95% CI, 0.99–1.3). When fibrinogen was performed as a quartile for analysis, a positive association between them was shown even after adjustment for potential confounders. Compared to participants with low fibrinogen in quartile 1 (<2.40 g/L), the adjusted OR values for fibrinogen and advanced colorectal adenoma in quartile 2 (2.40–2.75 g/L), quartile 3 (2.76–3.15 g/L), and quartile 4 (≥3.16 g/L) were 1.03 (95% CI: 0.76–1.41, *p* = 0.834), 1.37 (95% CI:1.01–1.85, *p* = 0.04), and 1.43 (95% CI: 1.06–1.96, *p* = 0.019; Table 2), respectively. The quartile 3 and quartile 4 groups (≥2.76 g/L) had the higher advanced colorectal adenoma incidence. To further explore the association between fibrinogen and advanced colorectal adenoma, smooth curve fitting was performed, and the results present a linear association between them (only 99% of the data was shown, *P* for non-linearity: 0.536, Figure 2).

**Table 2:** Multivariable logistic regression analyses of fibrinogen and advanced colorectal adenoma.

Variable	Event, *n* (%)	Crude model		Model I		Model II	
		OR (95%CI)	*P*-value	OR (95%CI)	*P*-value	OR (95%CI)	*P*-value
FIB, g/L	566/3738 (15.1)	1.36 (1.21–1.53)	<0.001	1.17 (1.03–1.33)	0.017	1.14 (0.99–1.3)	0.072
FIB quartile, g/L							
Q1 (<2.4)	107/906 (11.8)	1 (Reference)		1 (Reference)		1 (Reference)	
Q2 (2.4–2.75)	115/960 (12)	1.02 (0.77–1.35)	0.91	1.03 (0.76–1.4)	0.827	1.03 (0.76–1.41)	0.834
Q3 (2.76–3.15)	158/927 (17)	1.53 (1.18–2)	0.002	1.41 (1.05–1.89)	0.021	1.37 (1.01–1.85)	0.04
Q4 (≥3.16)	186/945 (19.7)	1.83 (1.41–2.37)	<0.001	1.47 (1.1–1.95)	0.008	1.43 (1.06–1.94)	0.019
*P* for trend			<0.001		0.001		0.005

Q, quartiles; OR, odds ratio; CI, confidence interval; FIB, fibrinogen; ALB, albumin; ALP, alkaline phosphatase; CREA, creatinine; PLT, platelets; APTT, activated partial thromboplastin time; DM, diabetes mellitus.

Crude model: no other covariates were adjusted.

Model I: adjusted for sex and age.

Model II: adjusted for sex, age, hypertension, DM, APTT, PLT, CREA, ALP, and ALB.

### 3.3. Sensitivity analysis

**Figure 3:**
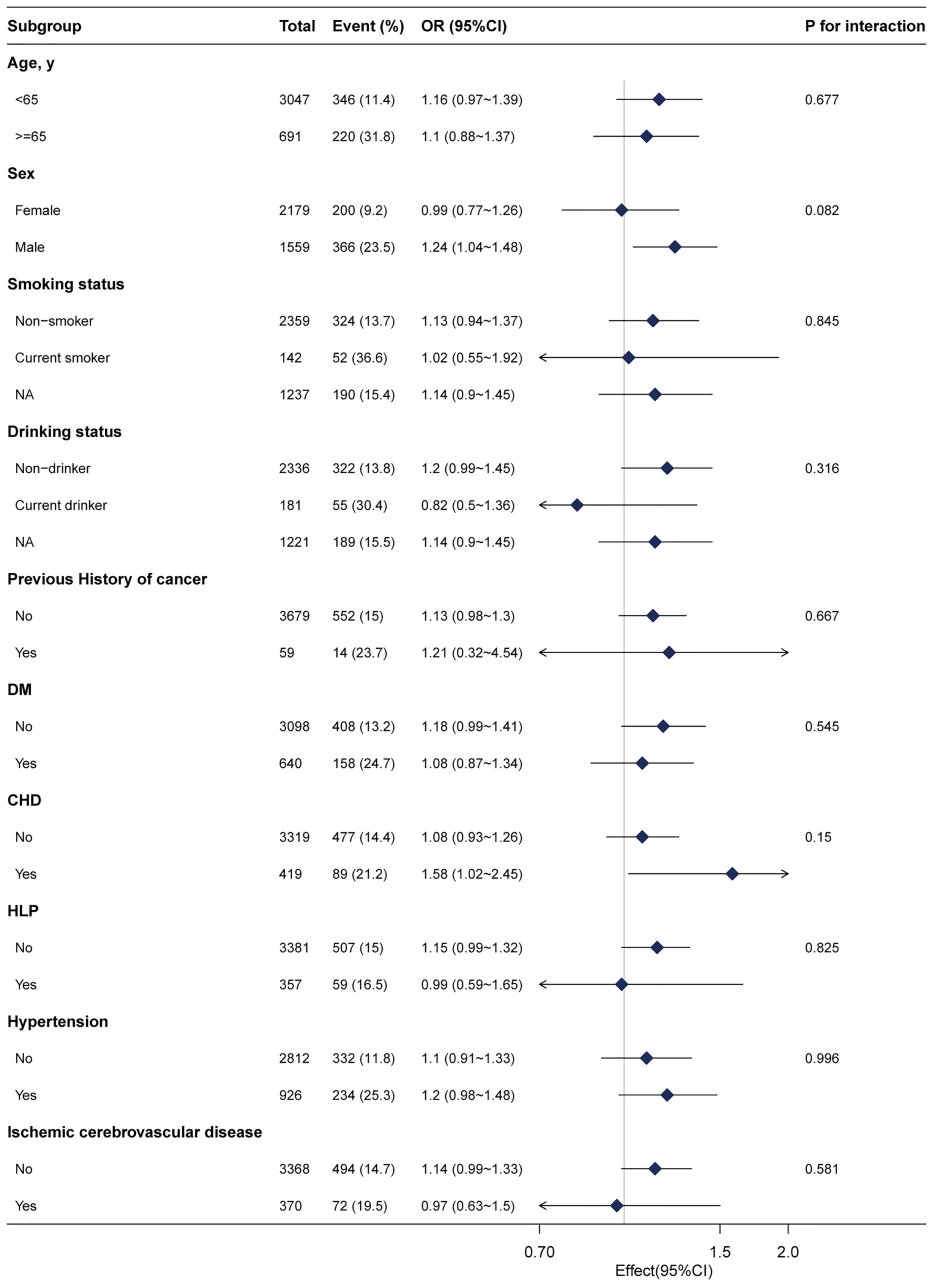
Subgroup analyses of the fibrinogen and advanced colorectal adenoma among inpatients. Except for the stratification component itself, each stratification factor was adjusted for age, sex, albumin, alkaline phosphatase, creatinine, platelets, activated partial thromboplastin time, hypertension, and diabetes mellitus. NA, not recorded.

The subgroup analyses were conducted to explore the relationship between fibrinogen and advanced colorectal adenoma among different layers. None of the variables, including age (<65 years, or ≥ 65 years), sex (male and female), smoking status, drinking status, history of cancer, DM, CHD, HLP, hypertension, ischemic cerebrovascular disease, significantly affected the relationship between fibrinogen and advanced colorectal adenoma (Figure 3). In subgroup analyses, we performed more indicators that have a significant difference in Table 1 (Supplementary Table S2). The results were stable as well.

In addition, sensitivity analysis was performed using the complete cases. All patients with missing data were excluded, and 3,738 individuals were left. And the association between fibrinogen and advanced colorectal adenoma remained stable [Model I: OR (95% CI), 1.17 (1.02–1.35); Model II: OR (95% CI), 1.13 (0.98–1.37)]. When serum GGT as a categorized variable, compared with individuals with lower fibrinogen in quartile 1 (<2.40 g/L), the adjusted OR values for fibrinogen and advanced colorectal adenoma in quartile 2 (2.40–2.74 g/L), quartile 3 (2.75–3.15 g/L), and quartile 4 (≥3.16 g/L) were 1.02 (95% CI: 0.73–1.42, *p* = 0.903), 1.39 (95% CI:1.01–1.9, *p* = 0.042), and 1.41 (95% CI: 1.02–1.94, *p* = 0.036; Model II, Supplementary Table SI), respectively. Furthermore, we used the fibrinogen-Z score in the multivariable logistic regression analyses (Supplementary Table S3) and found that with an elevated FIB-Z Score, the risk of advanced colorectal adenoma increased. These findings indicated the results were stable.

## 4. Discussion

This study explored the association between fibrinogen and advanced colorectal adenoma among inpatients. The fibrinogen was independently associated with advanced colorectal adenoma with linear association curves among inpatients. The present study found that the incidence of advanced colorectal adenoma increased as fibrinogen increased. The relationship between fibrinogen and advanced colorectal adenoma was stable between layers.

To our knowledge, there are no clinical studies demonstrating the correlation between fibrinogen and advanced adenoma. However, some literature may substantiate the relationship between them. Several studies have analyzed the plasma proteome of patients with advanced adenoma or colorectal cancer and found elevated fibrinogen α and β chains in the adenoma-cancer sequence, suggesting that fibrinogen may play a critical role in tumor progression (27–29). In addition, only two studies have associated plasma fibrinogen levels with CRC. Kristine et al. found elevated fibrinogen to be associated with an increased risk of CRC in 84,000 subjects, and this relationship was greatest during the early follow-up years. In addition, the authors suggest that the possible role of fibrinogen in CRC may be interpreted through its part in the inflammatory reaction (30). This finding supports the chronic low-level inflammation as a latent contributor of cancer development (30, 31). A nested case-cohort study indicated that fibrinogen (≥400 mg/dl) was positively correlated with CRC, suggesting that it may be a latent biomarker of CRC and supporting the “common soil” hypothesis (32). Advanced adenoma, a precursor of CRC, also showed an increased risk associated with elevated fibrinogen levels in our study. Complements the evidence that fibrinogen was positively associated with advanced adenomas.

Fibrinogen in the adenoma-carcinoma progression can be interpreted in terms of both hemostatic and inflammatory factors, and several hypotheses have been proposed. (i) Fibrinogen can bind growth factors (33), and therefore, fibrinogen residing on the cell matrix may act as a reservoir to control the bioavailability and accessibility of growth factors, and affect cancer cell proliferation, inhibit apoptosis, angiogenesis, and metastasis (34). (ii) Fibrinogen can bind to several types of cells. Fibrinogen-mediated cell bridging can exert traction for adhesion, shape change, motility, and invasion potential of cancer cells (35). (iii) Fibrinogen contributes to the protection of neoplastic cells from natural killer cell cytotoxicity through the interaction of β3 integrin’s with platelets, thus allowing escape from host immune surveillance (36). (iv) Fibrinogen is a critical regulator of inflammation in diseases, including cancer (17), and may have a role in cellular signaling by interaction with cellular receptors (37). Such interactions may promote inflammation, angiogenesis, and cell proliferation (33). Overall, fibrinogen effects and favors the adenoma-carcinoma progression, whether as an inflammatory or hemostatic factor.

Our study also had several limitations. First, the present research had limitations inherent to retrospective studies. We collected the participant’s data from the laboratory information system and electronic medical records, there were missing data for some indicators. However, we performed a sensitivity analysis and found that the results were stable with or without multiple imputations of the data. Second, in a present retrospective study, we did not collect repeated measurements for fibrinogen, which may not represent their long-term levels and their association with advanced colorectal adenoma. Third, even though multivariable logistic regression, subgroup analyses, and sensitivity analysis were performed, residual confounding effects from unmeasured or unknown factors could not be completely excluded. Finally, the findings were analyzed in a single-center database from China, and the generalizability may be limited for populations with different demographic characteristics.

Studying risk factors for advanced colorectal adenoma could help develop more comprehensive and non-invasive screening recommendations and possibly provide a clearer understanding of the mechanism of colon cancer. Studies on the epidemiology of advanced adenomas may be crucial in revealing why only a fraction of common adenomas progresses to CRC.

## 5. Conclusion

A linear association between fibrinogen and advanced colorectal adenoma was found among inpatients. With an increase in plasma fibrinogen, the risk of advanced adenoma increased. These findings provide evidence that fibrinogen may be a potential high-risk factor for colorectal screening.

## Data availability statement

The raw data supporting the conclusions of this article will be made available by the authors, without undue reservation.

## Ethics statement

The studies involving human participants were reviewed and approved by Ethics Review Board of Shijiazhuang Traditional Chinese Medicine Hospital (NO.20220919029). Written informed consent for participation was not required for this study in accordance with the national legislation and the institutional requirements.

## Author contributions

HW and HuZ conceived and designed the study and wrote and revised the manuscript. HW, XC, PM, JL, ZW, TZ, and HaZ collected and analyzed the data. All authors contributed to the article and approved the submitted version.

## Funding

This work was supported by the Hebei Administration of Traditional Chinese Medicine (grant number 2023145).

## Conflict of interest

The authors declare that the research was conducted in the absence of any commercial or financial relationships that could be construed as a potential conflict of interest.

## Publisher’s note

All claims expressed in this article are solely those of the authors and do not necessarily represent those of their affiliated organizations, or those of the publisher, the editors and the reviewers. Any product that may be evaluated in this article, or claim that may be made by its manufacturer, is not guaranteed or endorsed by the publisher.
