# Supplementary material for: Relationship between fibrinogen level and advanced colorectal adenoma among inpatients: A retrospective case-control study
Source: Front Med (Lausanne). 2023 Mar 16;10:1140185. doi: 10.3389/fmed.2023.1140185 (PMC10061582; doi:10.3389/fmed.2023.1140185)
Supplement: Supplementary file 1 [file Table_1.docx]

**Table S1** Multivariable logistic regression analyses of serum gamma-glutamyl transferase and advanced colorectal adenoma (before multiple imputations, n=3354).

| Variable | Event, n (%) | Crude model | |  | Model I | |  | Model II | |
| --- | --- | --- | --- | --- | --- | --- | --- | --- | --- |
|  |  | OR (95% CI) | *P* value |  | OR (95% CI) | *P* value |  | OR (95%CI) | *P* value |
| FIB, g/L | 504/3354(15) | 1.36 (1.2~1.54) | <0.001 |  | 1.17 (1.02~1.35) | 0.022 |  | 1.13 (0.98~1.31) | 0.097 |
| FIB quartile, g/L | |  |  |  |  |  |  |  |  |
| Q1 (<2.4) | 94/814(11.5) | 1(Reference) |  |  | 1(Reference) |  |  | 1(Reference) |  |
| Q2 (2.4-2.74) | 98/829(11.8) | 1.03 (0.76~1.39) | 0.863 |  | 1.03 (0.74~1.42) | 0.879 |  | 1.02 (0.73~1.42) | 0.903 |
| Q3 (2.75-3.15) | 151/870(17.4) | 1.61 (1.22~2.12) | 0.001 |  | 1.44 (1.06~1.96) | 0.021 |  | 1.39 (1.01~1.9) | 0.042 |
| Q4 (≥3.16) | 161/841(19.1) | 1.81 (1.38~2.39) | <0.001 |  | 1.46 (1.08~1.98) | 0.014 |  | 1.41 (1.02~1.94) | 0.036 |
| P for trend | |  | <0.001 |  |  | 0.003 |  |  | 0.01 |

Abbreviations: Q, quartiles; OR, odds ratio; CI, confidence interval; FIB, fibrinogen; ALB, albumin; ALP, alkaline phosphatase; CREA, creatinine; PLT, platelets; APTT, activated partial thromboplastin time; DM, diabetes mellitus.

Crude model: no other covariates were adjusted.

Model I: adjusted for sex and age.

Model II: adjusted for sex, age, hypertension, DM, APTT, PLT, CREA, ALP, ALB.
