# Supplementary material for: Relationship between fibrinogen level and advanced colorectal adenoma among inpatients: A retrospective case-control study
Source: Front Med (Lausanne). 2023 Mar 16;10:1140185. doi: 10.3389/fmed.2023.1140185 (PMC10061582; doi:10.3389/fmed.2023.1140185)
Supplement: Supplementary file 2 [file Table_2.docx]

**Table S2** Subgroup analyses between fibrinogen and advanced colorectal adenoma.

| **Subgroups** | **Total, n** | **Event, n (%)** | **OR (95% CI）** | ***P* value** | ***P* for interaction** |
| --- | --- | --- | --- | --- | --- |
| Age, y |  |  |  |  |  |
| <65 | 3047 | 346 (11.4) | 1.16 (0.97~1.39) | 0.104 | 0.677 |
| ≥65 | 691 | 220 (31.8) | 1.1 (0.88~1.37) | 0.397 |  |
| Sex |  |  |  |  |  |
| Female | 2179 | 200 (9.2) | 0.99 (0.77~1.26) | 0.928 | 0.082 |
| Male | 1559 | 366 (23.5) | 1.24 (1.04~1.48) | 0.016 |  |
| Smoking status | |  |  |  |  |
| Non-smoker | 2359 | 324 (13.7) | 1.13 (0.94~1.37) | 0.185 | 0.845 |
| Current smoker | 142 | 52 (36.6) | 1.02 (0.55~1.92) | 0.94 |  |
| NA | 1237 | 190 (15.4) | 1.14 (0.9~1.45) | 0.266 |  |
| Drinking status | |  |  |  |  |
| Non-drinker | 2336 | 322 (13.8) | 1.2 (0.99~1.45) | 0.062 | 0.316 |
| Current drinker | 181 | 55 (30.4) | 0.82 (0.5~1.36) | 0.446 |  |
| NA | 1221 | 189 (15.5) | 1.14 (0.9~1.45) | 0.27 |  |
| Previous History of cancer | | |  |  |  |
| No | 3679 | 552 (15) | 1.13 (0.98~1.3) | 0.082 | 0.667 |
| Yes | 59 | 14 (23.7) | 1.21 (0.32~4.54) | 0.775 |  |
| DM |  |  |  |  |  |
| No | 3098 | 408 (13.2) | 1.18 (0.99~1.41) | 0.073 | 0.545 |
| Yes | 640 | 158 (24.7) | 1.08 (0.87~1.34) | 0.462 |  |
| CHD |  |  |  |  |  |
| No | 3319 | 477 (14.4) | 1.08 (0.93~1.26) | 0.287 | 0.15 |
| Yes | 419 | 89 (21.2) | 1.58 (1.02~2.45) | 0.04 |  |
| HLP |  |  |  |  |  |
| No | 3381 | 507 (15) | 1.15 (0.99~1.32) | 0.063 | 0.825 |
| Yes | 357 | 59 (16.5) | 0.99 (0.59~1.65) | 0.963 |  |
| Hypertension | |  |  |  |  |
| No | 2812 | 332 (11.8) | 1.1 (0.91~1.33) | 0.318 | 0.996 |
| Yes | 926 | 234 (25.3) | 1.2 (0.98~1.48) | 0.083 |  |
| Ischemic cerebrovascular disease | | |  |  |  |
| No | 3368 | 494 (14.7) | 1.14 (0.99~1.33) | 0.075 | 0.581 |
| Yes | 370 | 72 (19.5) | 0.97 (0.63~1.5) | 0.896 |  |
| Urea, mmol/L |  |  |  |  |  |
| <4.6 | 1847 | 215 (11.6) | 1.11 (0.89~1.39) | 0.344 | 0.969 |
| ≥4.6 | 1891 | 351 (18.6) | 1.17 (0.98~1.4) | 0.089 |  |
| GLU, mmol/L |  |  |  |  |  |
| <5.54 | 1859 | 198 (10.7) | 1.15 (0.9~1.46) | 0.264 | 0.514 |
| ≥5.54 | 1879 | 368 (19.6) | 1.12 (0.94~1.32) | 0.205 |  |
| UA, μmol/L |  |  |  |  |  |
| <292 | 1861 | 225 (12.1) | 0.98 (0.78~1.24) | 0.891 | 0.164 |
| ≥292 | 1877 | 341 (18.2) | 1.25 (1.04~1.5) | 0.016 |  |

Abbreviations: CHD, coronary heart disease; HLP, hyperlipemia; DM, diabetes mellitus; GLU, glucose; UA, uric acid. Adjusted for sex, age, hypertension, DM, APTT, PLT, CREA, ALP, and ALB.
