# Supplementary material for: Relationship between fibrinogen level and advanced colorectal adenoma among inpatients: A retrospective case-control study
Source: Front Med (Lausanne). 2023 Mar 16;10:1140185. doi: 10.3389/fmed.2023.1140185 (PMC10061582; doi:10.3389/fmed.2023.1140185)
Supplement: Supplementary file 3 [file Table_3.docx]

**Table S3** Multivariable logistic regression analyses of the fibrinogen-Z score and advanced colorectal adenoma.

| Variable | Event, n (%) | Crude model | |  | Model I | |  | Model II | |
| --- | --- | --- | --- | --- | --- | --- | --- | --- | --- |
|  |  | OR (95%CI) | *P* value |  | OR (95%CI) | *P* value |  | OR (95%CI) | *P* value |
| FIB-Z Score | 566/3738 (15.1) | 1.23 (1.14~1.34) | <0.001 |  | 1.11 (1.02~1.22) | 0.017 |  | 1.1 (0.99~1.21) | 0.066 |
| FIB-Z Score, quartile | |  |  |  |  |  |  |  |  |
| Q1 (<-0.660) | 107/906 (11.8) | 1(Reference) |  |  | 1(Reference) |  |  | 1(Reference) |  |
| Q2 (-0.660–-0.135) | 115/960 (12) | 1.02 (0.77~1.35) | 0.91 |  | 1.03 (0.76~1.4) | 0.827 |  | 1.05 (0.76~1.45) | 0.77 |
| Q3 (-0.136–-0.446) | 158/927 (17) | 1.53 (1.18~2) | 0.002 |  | 1.41 (1.05~1.89) | 0.021 |  | 1.39 (1.02~1.91) | 0.04 |
| Q4 (≥0.447) | 186/945 (19.7) | 1.83 (1.41~2.37) | <0.001 |  | 1.47 (1.1~1.95) | 0.008 |  | 1.45 (1.05~1.98) | 0.022 |
| *P* for trend |  |  | <0.001 |  |  | 0.001 |  |  | 0.028 |

Abbreviations: Q, quartiles; OR, odds ratio; CI, confidence interval; FIB, fibrinogen; ALB, albumin; ALP, alkaline phosphatase; CREA, creatinine; PLT, platelets; APTT, activated partial thromboplastin time; DM, diabetes mellitus.

Crude model: no other covariates were adjusted.

Model I: adjusted for sex and age.

Model II: adjusted for sex, age, hypertension, DM, APTT, PLT, CREA, ALP, and ALB.
